# Carbolic Acid

**Published:** 1869-08-15

**Authors:** 


### *Carbolic Acid.*

We are agreeable impressed by the statement of a correspondent of one of our homœopathic exchanges, that there has been a "proving" of this important substance by its internal exhibition. "Two hundred and eighty-four symptoms have been elicited by the five provers, and conveniently arranged for reference." A pamphlet has been published on the subject, which can be obtained at Keen & Cooke's. The correspondent well remarks that: "The results attained should be an incentive to more thorough and extended provings of the drug."

We think it should. Meanwhile will this Lord help us?

